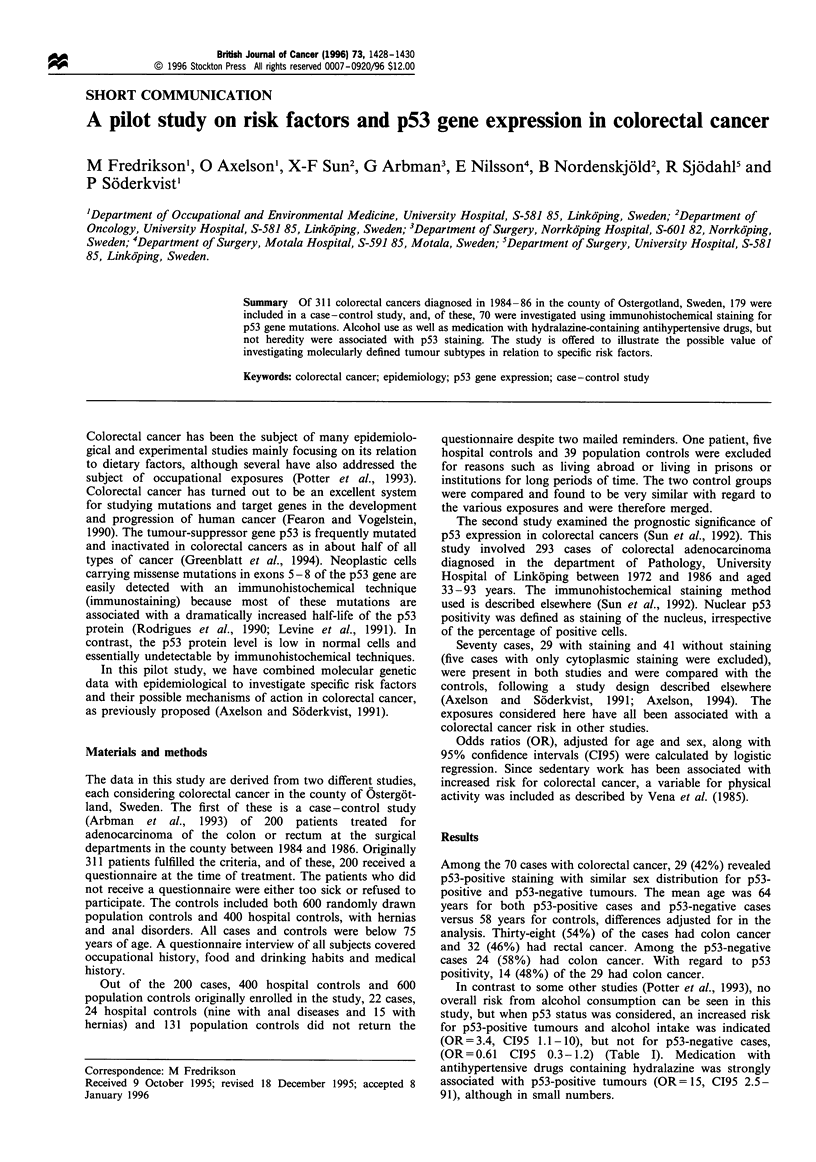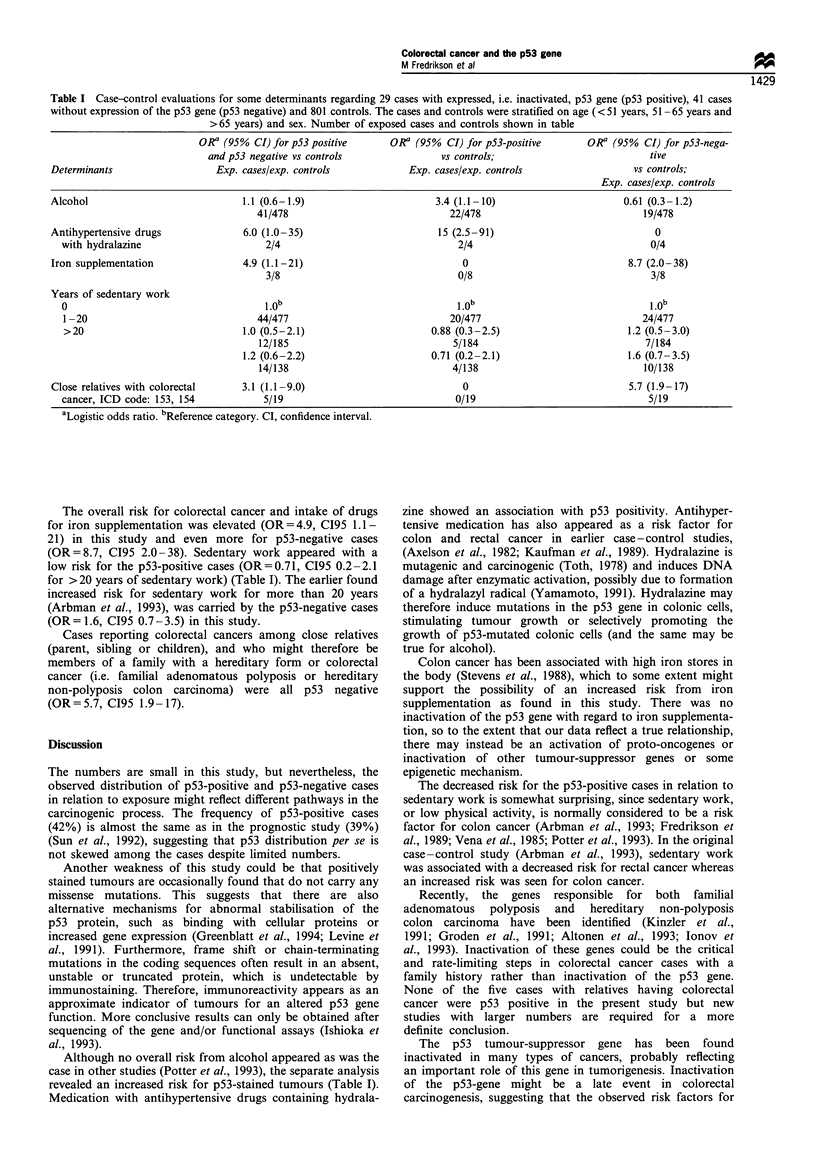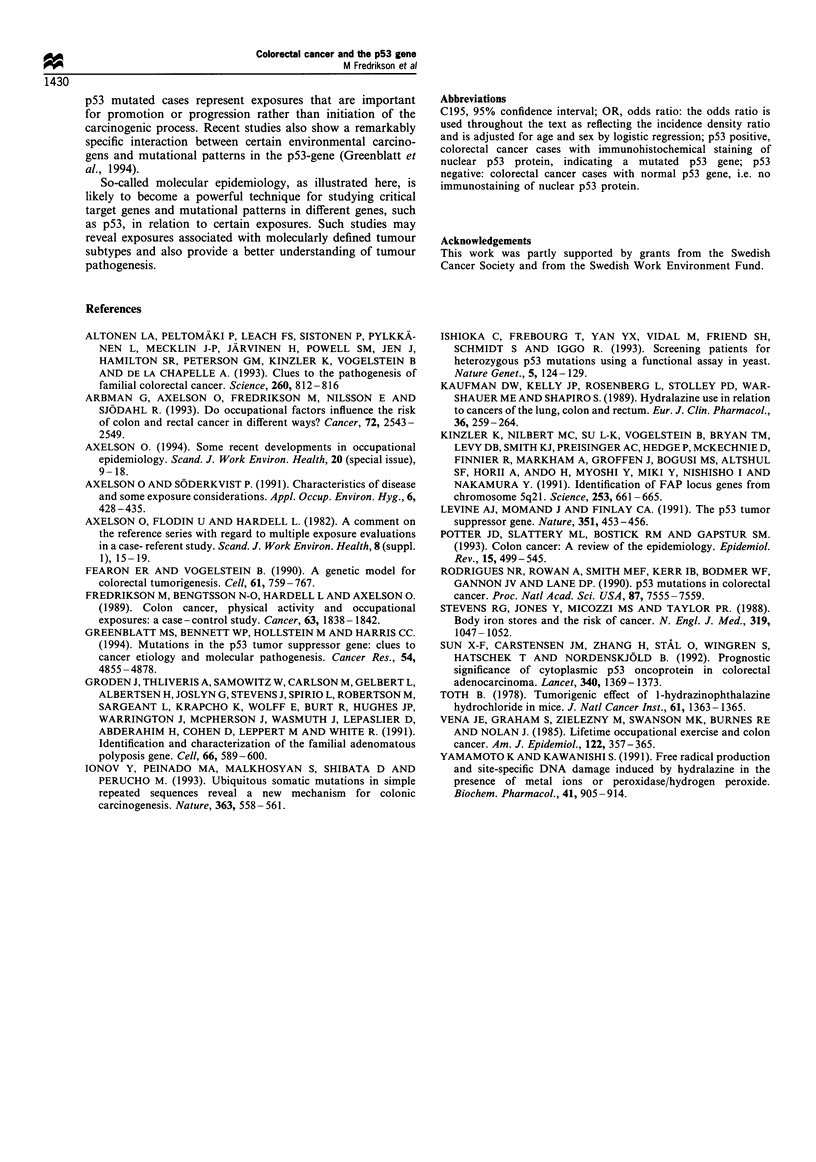# A pilot study on risk factors and p53 gene expression in colorectal cancer.

**DOI:** 10.1038/bjc.1996.271

**Published:** 1996-06

**Authors:** M. Fredrikson, O. Axelson, X. F. Sun, G. Arbman, E. Nilsson, B. Nordenskjöld, R. Sjödahl, P. Söderkvist

**Affiliations:** Department of Occupational and Environmental Medicine, University Hospital, Linköping, Sweden.

## Abstract

Of 311 colorectal cancers diagnosed in 1984-86 in the county of Ostergotland, Sweden, 179 were included in a case-control study, and, of these, 70 were investigated using immunohistochemical staining for p53 gene mutations. Alcohol use as well as medication with hydralazine-containing antihypertensive drugs, but not heredity were associated with p53 staining. The study is offered to illustrate the possible value of investigating molecularly defined tumour subtypes in relation to specific risk factors.


British Journal of Cancer (1996) 73, 1428-1430
© 1996 Stockton Press All rights reserved 0007-0920/96 $12.00

SHORT COMMUNICATION

### A pilot study on risk factors and p53 gene expression in colorectal cancer

M Fredrikson[1], O Axelson[1], X-F Sun[2], G Arbman[3], E Nilsson[4], B Nordenskjöld[2], R Sjödahl[5] and P Söderkvist[1]

[1]*Department of Occupational and Environmental Medicine, University Hospital, S-581 85, Linköping, Sweden;* [2]*Department of Oncology, University Hospital, S-581 85, Linköping, Sweden;* [3]*Department of Surgery, Norrköping Hospital, S-601 82, Norrköping, Sweden;* [4]*Department of Surgery, Motala Hospital, S-591 85, Motala, Sweden;* [5]*Department of Surgery, University Hospital, S-581 85, Linköping, Sweden.*

**Summary** Of 311 colorectal cancers diagnosed in 1984–86 in the county of Ostergotland, Sweden, 179 were included in a case–control study, and, of these, 70 were investigated using immunohistochemical staining for p53 gene mutations. Alcohol use as well as medication with hydralazine-containing antihypertensive drugs, but not heredity were associated with p53 staining. The study is offered to illustrate the possible value of investigating molecularly defined tumour subtypes in relation to specific risk factors.

**Keywords:** colorectal cancer; epidemiology; p53 gene expression; case–control study

Colorectal cancer has been the subject of many epidemiological and experimental studies mainly focusing on its relation to dietary factors, although several have also addressed the subject of occupational exposures (Potter *et al.*, 1993). Colorectal cancer has turned out to be an excellent system for studying mutations and target genes in the development and progression of human cancer (Fearon and Vogelstein, 1990). The tumour-suppressor gene p53 is frequently mutated and inactivated in colorectal cancers as in about half of all types of cancer (Greenblatt *et al.*, 1994). Neoplastic cells carrying missense mutations in exons 5–8 of the p53 gene are easily detected with an immunohistochemical technique (immunostaining) because most of these mutations are associated with a dramatically increased half-life of the p53 protein (Rodrigues *et al.*, 1990; Levine *et al.*, 1991). In contrast, the p53 protein level is low in normal cells and essentially undetectable by immunohistochemical techniques.

In this pilot study, we have combined molecular genetic data with epidemiological to investigate specific risk factors and their possible mechanisms of action in colorectal cancer, as previously proposed (Axelson and Söderkvist, 1991).

### Materials and methods

The data in this study are derived from two different studies, each considering colorectal cancer in the county of Östergötland, Sweden. The first of these is a case–control study (Arbman *et al.*, 1993) of 200 patients treated for adenocarcinoma of the colon or rectum at the surgical departments in the county between 1984 and 1986. Originally 311 patients fulfilled the criteria, and of these, 200 received a questionnaire at the time of treatment. The patients who did not receive a questionnaire were either too sick or refused to participate. The controls included both 600 randomly drawn population controls and 400 hospital controls, with hernias and anal disorders. All cases and controls were below 75 years of age. A questionnaire interview of all subjects covered occupational history, food and drinking habits and medical history.

Out of the 200 cases, 400 hospital controls and 600 population controls originally enrolled in the study, 22 cases, 24 hospital controls (nine with anal diseases and 15 with hernias) and 131 population controls did not return the questionnaire despite two mailed reminders. One patient, five hospital controls and 39 population controls were excluded for reasons such as living abroad or living in prisons or institutions for long periods of time. The two control groups were compared and found to be very similar with regard to the various exposures and were therefore merged.

The second study examined the prognostic significance of p53 expression in colorectal cancers (Sun *et al.*, 1992). This study involved 293 cases of colorectal adenocarcinoma diagnosed in the department of Pathology, University Hospital of Linköping between 1972 and 1986 and aged 33–93 years. The immunohistochemical staining method used is described elsewhere (Sun *et al.*, 1992). Nuclear p53 positivity was defined as staining of the nucleus, irrespective of the percentage of positive cells.

Seventy cases, 29 with staining and 41 without staining (five cases with only cytoplasmic staining were excluded), were present in both studies and were compared with the controls, following a study design described elsewhere (Axelson and Söderkvist, 1991; Axelson, 1994). The exposures considered here have all been associated with a colorectal cancer risk in other studies.

Odds ratios (OR), adjusted for age and sex, along with 95% confidence intervals (CI95) were calculated by logistic regression. Since sedentary work has been associated with increased risk for colorectal cancer, a variable for physical activity was included as described by Vena *et al.* (1985).

### Results

Among the 70 cases with colorectal cancer, 29 (42%) revealed p53-positive staining with similar sex distribution for p53-positive and p53-negative tumours. The mean age was 64 years for both p53-positive cases and p53-negative cases versus 58 years for controls, differences adjusted for in the analysis. Thirty-eight (54%) of the cases had colon cancer and 32 (46%) had rectal cancer. Among the p53-negative cases 24 (58%) had colon cancer. With regard to p53 positivity, 14 (48%) of the 29 had colon cancer.

In contrast to some other studies (Potter *et al.*, 1993), no overall risk from alcohol consumption can be seen in this study, but when p53 status was considered, an increased risk for p53-positive tumours and alcohol intake was indicated (OR=3.4, CI95 1.1–10), but not for p53-negative cases, (OR=0.61 CI95 0.3–1.2) (Table I). Medication with antihypertensive drugs containing hydralazine was strongly associated with p53-positive tumours (OR=15, CI95 2.5–91), although in small numbers.

Correspondence: M Fredrikson
Received 9 October 1995; revised 18 December 1995; accepted 8 January 1996

**Table I** Case–control evaluations for some determinants regarding 29 cases with expressed, i.e. inactivated, p53 gene (p53 positive), 41 cases without expression of the p53 gene (p53 negative) and 801 controls. The cases and controls were stratified on age (<51 years, 51–65 years and >65 years) and sex. Number of exposed cases and controls shown in table

| *Determinants* | *OR[a] (95% CI) for p53 positive and p53 negative vs controls Exp. cases/exp. controls* | *OR[a] (95% CI) for p53-positive vs controls; Exp. cases/exp. controls* | *OR[a] (95% CI) for p53-negative vs controls; Exp. cases/exp. controls* |
|---|---|---|---|
| Alcohol | 1.1 (0.6–1.9) | 3.4 (1.1–10) | 0.61 (0.3–1.2) |
| | 41/478 | 22/478 | 19/478 |
| Antihypertensive drugs | 6.0 (1.0–35) | 15 (2.5–91) | 0 |
| with hydralazine | 2/4 | 2/4 | 0/4 |
| Iron supplementation | 4.9 (1.1–21) | 0 | 8.7 (2.0–38) |
| | 3/8 | 0/8 | 3/8 |
| Years of sedentary work | | | |
| 0 | 1.0[b] | 1.0[b] | 1.0[b] |
| 1–20 | 44/477 | 20/477 | 24/477 |
| >20 | 1.0 (0.5–2.1) | 0.88 (0.3–2.5) | 1.2 (0.5–3.0) |
| | 12/185 | 5/184 | 7/184 |
| | 1.2 (0.6–2.2) | 0.71 (0.2–2.1) | 1.6 (0.7–3.5) |
| | 14/138 | 4/138 | 10/138 |
| Close relatives with colorectal | 3.1 (1.1–9.0) | 0 | 5.7 (1.9–17) |
| cancer, ICD code: 153, 154 | 5/19 | 0/19 | 5/19 |

[a]Logistic odds ratio. [b]Reference category. CI, confidence interval.

The overall risk for colorectal cancer and intake of drugs for iron supplementation was elevated (OR = 4.9, CI95 1.1–21) in this study and even more for p53-negative cases (OR = 8.7, CI95 2.0–38). Sedentary work appeared with a low risk for the p53-positive cases (OR = 0.71, CI95 0.2–2.1 for >20 years of sedentary work) (Table I). The earlier found increased risk for sedentary work for more than 20 years (Arbman *et al*., 1993), was carried by the p53-negative cases (OR = 1.6, CI95 0.7–3.5) in this study.

Cases reporting colorectal cancers among close relatives (parent, sibling or children), and who might therefore be members of a family with a hereditary form or colorectal cancer (i.e. familial adenomatous polyposis or hereditary non-polyposis colon carcinoma) were all p53 negative (OR = 5.7, CI95 1.9–17).

### Discussion

The numbers are small in this study, but nevertheless, the observed distribution of p53-positive and p53-negative cases in relation to exposure might reflect different pathways in the carcinogenic process. The frequency of p53-positive cases (42%) is almost the same as in the prognostic study (39%) (Sun *et al*., 1992), suggesting that p53 distribution *per se* is not skewed among the cases despite limited numbers.

Another weakness of this study could be that positively stained tumours are occasionally found that do not carry any missense mutations. This suggests that there are also alternative mechanisms for abnormal stabilisation of the p53 protein, such as binding with cellular proteins or increased gene expression (Greenblatt *et al*., 1994; Levine *et al*., 1991). Furthermore, frame shift or chain-terminating mutations in the coding sequences often result in an absent, unstable or truncated protein, which is undetectable by immunostaining. Therefore, immunoreactivity appears as an approximate indicator of tumours for an altered p53 gene function. More conclusive results can only be obtained after sequencing of the gene and/or functional assays (Ishioka *et al*., 1993).

Although no overall risk from alcohol appeared as was the case in other studies (Potter *et al*., 1993), the separate analysis revealed an increased risk for p53-stained tumours (Table I). Medication with antihypertensive drugs containing hydralazine showed an association with p53 positivity. Antihypertensive medication has also appeared as a risk factor for colon and rectal cancer in earlier case–control studies, (Axelson *et al*., 1982; Kaufman *et al*., 1989). Hydralazine is mutagenic and carcinogenic (Toth, 1978) and induces DNA damage after enzymatic activation, possibly due to formation of a hydralazyl radical (Yamamoto, 1991). Hydralazine may therefore induce mutations in the p53 gene in colonic cells, stimulating tumour growth or selectively promoting the growth of p53-mutated colonic cells (and the same may be true for alcohol).

Colon cancer has been associated with high iron stores in the body (Stevens *et al*., 1988), which to some extent might support the possibility of an increased risk from iron supplementation as found in this study. There was no inactivation of the p53 gene with regard to iron supplementation, so to the extent that our data reflect a true relationship, there may instead be an activation of proto-oncogenes or inactivation of other tumour-suppressor genes or some epigenetic mechanism.

The decreased risk for the p53-positive cases in relation to sedentary work is somewhat surprising, since sedentary work, or low physical activity, is normally considered to be a risk factor for colon cancer (Arbman *et al*., 1993; Fredrikson *et al*., 1989; Vena *et al*., 1985; Potter *et al*., 1993). In the original case–control study (Arbman *et al*., 1993), sedentary work was associated with a decreased risk for rectal cancer whereas an increased risk was seen for colon cancer.

Recently, the genes responsible for both familial adenomatous polyposis and hereditary non-polyposis colon carcinoma have been identified (Kinzler *et al*., 1991; Groden *et al*., 1991; Altonen *et al*., 1993; Ionov *et al*., 1993). Inactivation of these genes could be the critical and rate-limiting steps in colorectal cancer cases with a family history rather than inactivation of the p53 gene. None of the five cases with relatives having colorectal cancer were p53 positive in the present study but new studies with larger numbers are required for a more definite conclusion.

The p53 tumour-suppressor gene has been found inactivated in many types of cancers, probably reflecting an important role of this gene in tumorigenesis. Inactivation of the p53-gene might be a late event in colorectal carcinogenesis, suggesting that the observed risk factors for p53 mutated cases represent exposures that are important for promotion or progression rather than initiation of the carcinogenic process. Recent studies also show a remarkably specific interaction between certain environmental carcinogens and mutational patterns in the p53-gene (Greenblatt *et al.*, 1994).

So-called molecular epidemiology, as illustrated here, is likely to become a powerful technique for studying critical target genes and mutational patterns in different genes, such as p53, in relation to certain exposures. Such studies may reveal exposures associated with molecularly defined tumour subtypes and also provide a better understanding of tumour pathogenesis.

### Abbreviations

C195, 95% confidence interval; OR, odds ratio: the odds ratio is used throughout the text as reflecting the incidence density ratio and is adjusted for age and sex by logistic regression; p53 positive, colorectal cancer cases with immunohistochemical staining of nuclear p53 protein, indicating a mutated p53 gene; p53 negative: colorectal cancer cases with normal p53 gene, i.e. no immunostaining of nuclear p53 protein.

### Acknowledgements

This work was partly supported by grants from the Swedish Cancer Society and from the Swedish Work Environment Fund.

### References

ALTONEN LA, PELTOMÄKI P, LEACH FS, SISTONEN P, PYLKKÄNEN L, MECKLIN J-P, JÄRVINEN H, POWELL SM, JEN J, HAMILTON SR, PETERSON GM, KINZLER K, VOGELSTEIN B AND DE LA CHAPELLE A. (1993). Clues to the pathogenesis of familial colorectal cancer. *Science*, **260**, 812-816

ARBMAN G, AXELSON O, FREDRIKSON M, NILSSON E AND SJÖDAHL R. (1993). Do occupational factors influence the risk of colon and rectal cancer in different ways? *Cancer*, **72**, 2543-2549.

AXELSON O. (1994). Some recent developments in occupational epidemiology. *Scand. J. Work Environ. Health*, **20** (special issue), 9-18.

AXELSON O AND SÖDERKVIST P. (1991). Characteristics of disease and some exposure considerations. *Appl. Occup. Environ. Hyg.*, **6**, 428-435.

AXELSON O, FLODIN U AND HARDELL L. (1982). A comment on the reference series with regard to multiple exposure evaluations in a case- referent study. *Scand. J. Work Environ. Health*, **8** (suppl. 1), 15-19.

FEARON ER AND VOGELSTEIN B. (1990). A genetic model for colorectal tumorigenesis. *Cell*, **61**, 759-767.

FREDRIKSON M, BENGTSSON N-O, HARDELL L AND AXELSON O. (1989). Colon cancer, physical activity and occupational exposures: a case-control study. *Cancer*, **63**, 1838-1842.

GREENBLATT MS, BENNETT WP, HOLLSTEIN M AND HARRIS CC. (1994). Mutations in the p53 tumor suppressor gene: clues to cancer etiology and molecular pathogenesis. *Cancer Res.*, **54**, 4855-4878.

GRODEN J, THLIVERIS A, SAMOWITZ W, CARLSON M, GELBERT L, ALBERTSEN H, JOSLYN G, STEVENS J, SPIRIO L, ROBERTSON M, SARGEANT L, KRAPCHO K, WOLFF E, BURT R, HUGHES JP, WARRINGTON J, MCPHERSON J, WASMUTH J, LEPASLIER D, ABDERAHIM H, COHEN D, LEPPERT M AND WHITE R. (1991). Identification and characterization of the familial adenomatous polyposis gene. *Cell*, **66**, 589-600.

IONOV Y, PEINADO MA, MALKHOSYAN S, SHIBATA D AND PERUCHO M. (1993). Ubiquitous somatic mutations in simple repeated sequences reveal a new mechanism for colonic carcinogenesis. *Nature*, **363**, 558-561.

ISHIOKA C, FREBOURG T, YAN YX, VIDAL M, FRIEND SH, SCHMIDT S AND IGGO R. (1993). Screening patients for heterozygous p53 mutations using a functional assay in yeast. *Nature Genet.*, **5**, 124-129.

KAUFMAN DW, KELLY JP, ROSENBERG L, STOLLEY PD, WARSHAUER ME AND SHAPIRO S. (1989). Hydralazine use in relation to cancers of the lung, colon and rectum. *Eur. J. Clin. Pharmacol.*, **36**, 259-264.

KINZLER K, NILBERT MC, SU L-K, VOGELSTEIN B, BRYAN TM, LEVY DB, SMITH KJ, PREISINGER AC, HEDGE P, MCKECHNIE D, FINNIER R, MARKHAM A, GROFFEN J, BOGUSI MS, ALTSHUL SF, HORII A, ANDO H, MYOSHI Y, MIKI Y, NISHISHO I AND NAKAMURA Y. (1991). Identification of FAP locus genes from chromosome 5q21. *Science*, **253**, 661-665.

LEVINE AJ, MOMAND J AND FINLAY CA. (1991). The p53 tumor suppressor gene. *Nature*, **351**, 453-456.

POTTER JD, SLATTERY ML, BOSTICK RM AND GAPSTUR SM. (1993). Colon cancer: A review of the epidemiology. *Epidemiol. Rev.*, **15**, 499-545.

RODRIGUES NR, ROWAN A, SMITH MEF, KERR IB, BODMER WF, GANNON JV AND LANE DP. (1990). p53 mutations in colorectal cancer. *Proc. Natl Acad. Sci. USA*, **87**, 7555-7559.

STEVENS RG, JONES Y, MICOZZI MS AND TAYLOR PR. (1988). Body iron stores and the risk of cancer. *N. Engl. J. Med.*, **319**, 1047-1052.

SUN X-F, CARSTENSEN JM, ZHANG H, STÅL O, WINGREN S, HATSCHEK T AND NORDENSKJÖLD B. (1992). Prognostic significance of cytoplasmic p53 oncoprotein in colorectal adenocarcinoma. *Lancet*, **340**, 1369-1373.

TOTH B. (1978). Tumorigenic effect of 1-hydrazinophthalazine hydrochloride in mice. *J. Natl Cancer Inst.*, **61**, 1363-1365.

VENA JE, GRAHAM S, ZIELEZNY M, SWANSON MK, BURNES RE AND NOLAN J. (1985). Lifetime occupational exercise and colon cancer. *Am. J. Epidemiol.*, **122**, 357-365.

YAMAMOTO K AND KAWANISHI S. (1991). Free radical production and site-specific DNA damage induced by hydralazine in the presence of metal ions or peroxidase/hydrogen peroxide. *Biochem. Pharmacol.*, **41**, 905-914.